# Improved Accuracy of Multidimensional Spatially‐Selective Saturation RF Pulses for Short‐Repetition‐Time Steady‐State Imaging

**DOI:** 10.1002/mrm.70573

**Published:** 2026-08-26

**Authors:** Yongli He, Douglas C. Noll, Jon‐Fredrik Nielsen

**Affiliations:** ^1^ Department of Biomedical Engineering University of Michigan Ann Arbor Michigan USA; ^2^ Applied Physics Program University of Michigan Ann Arbor Michigan USA; ^3^ Department of Radiology University of Michigan Ann Arbor Michigan USA

**Keywords:** inverse problem, RF pulse design, steady‐state imaging, tailored excitation

## Abstract

**Purpose:**

To introduce and evaluate a novel RF pulse design algorithm for multi‐dimensional spatially tailored excitation that is optimized for spoiled steady‐state gradient echo MRI sequences.

**Methods:**

Existing multi‐dimensional RF pulse design algorithms optimize RF and gradient waveforms using a loss function based on a single RF pulse application. These designs are poorly suited for steady‐state MRI sequences where RF pulses are spaced only tens of milliseconds apart, causing potentially large differences between the steady‐state magnetization and the target pattern. We propose a multidimensional RF pulse design algorithm with a loss function based on the steady‐state magnetization of a spoiled gradient‐echo sequence. Our algorithm, steady‐state AutoDiffPulses (SS‐ADP), extends the previously introduced ADP framework for joint RF and gradient pulse design. We evaluated design accuracy and RF energy in phantom and in vivo brain imaging in inner‐volume (IV) and outer‐volume (OV) saturation experiments. We further evaluated the potential of SS‐ADP to improve reduced FOV (rFOV) imaging.

**Results:**

SS‐ADP produced significantly more accurate steady‐state magnetization patterns compared to a conventional single‐excitation design (ADP), with significantly reduced RF energy, for $\mathrm{TR}≲0.2T_{1}$. Furthermore, utilizing SS‐ADP for outer‐volume suppression (OVS) substantially reduced reconstruction error in accelerated rFOV imaging compared to conventional ADP‐OVS.

**Conclusion:**

A joint RF and gradient pulse design algorithm accounting for steady‐state magnetization evolution significantly improves excitation accuracy and reduces SAR in short‐TR spoiled imaging with $\mathrm{TR}≲0.2T_{1}$.

## 1 Introduction

In MRI, it is often desirable to create spatially tailored radiofrequency (RF) pulses that excite only a certain subset of spins within some application‐dependent region‐of‐interest (ROI). For example, in reduced field‐of‐view (rFOV) imaging, the signal outside the ROI can be suppressed by a spatially selective pulse, which can reduce under‐sampling artifacts and improve spatio‐temporal resolution. Over the past decades, many groups have demonstrated the possibility to create 2D/3D selective excitations for this purpose [1, 2, 3, 4, 5, 6, 7, 8]. Most recently, Luo et al. [8] proposed an auto‐differentiation‐based framework called AutoDiffPulses (ADP) that jointly optimizes RF and gradient waveforms with the capability to design tailored pulses for arbitrary flip angles. The multidimensional tailored pulses by this framework are highly efficient, with duration as short as a few ms.

However, ADP has reduced performance in designing RF pulses for steady‐state (SS) imaging sequences. For example, in our simulation to be shown below (Section 3.2), the excitation normalized‐root‐mean‐square‐error (NRMSE) of the tailored pulse increased from ∼0.15 in the non‐SS sequence to ∼0.50 in the SS sequence. This phenomenon is due to the framework's inherent assumption that the sequence repetition time (TR) is long enough for the longitudinal magnetization (LM) to fully recover, whereas in SS imaging, the TR is very short ($\ll T_{1}$) and only partial recovery is achieved between RF pulse applications. In this work, we correct this model mismatch and make the ADP framework applicable to SS imaging. Specifically, we apply this approach to one common class of SS sequence, RF‐spoiled GRE (SPGR/FLASH/T1‐FFE), as an example, and derive the relationship between SS magnetization and the tailored RF pulse. Then we integrate this analysis into the original algorithm in Luo et al. [8]. We call our version of the algorithm SS‐AutoDiffPulses (SS‐ADP). We demonstrate that the SS‐ADP algorithm generates more accurate multidimensional spatially selective pulses with two specific design examples: outer‐volume suppression (OVS) and inner‐volume suppression (IVS). With the wide clinical and research use of SS sequences, we believe our new algorithm will be useful in many MRI applications.

## 2 Methods

### 2.1 Algorithm

Figure 1A shows the flowchart of the original ADP [8] framework and the proposed SS version. In this section, we first review the original ADP framework, then we introduce our main contribution.

**FIGURE 1:**
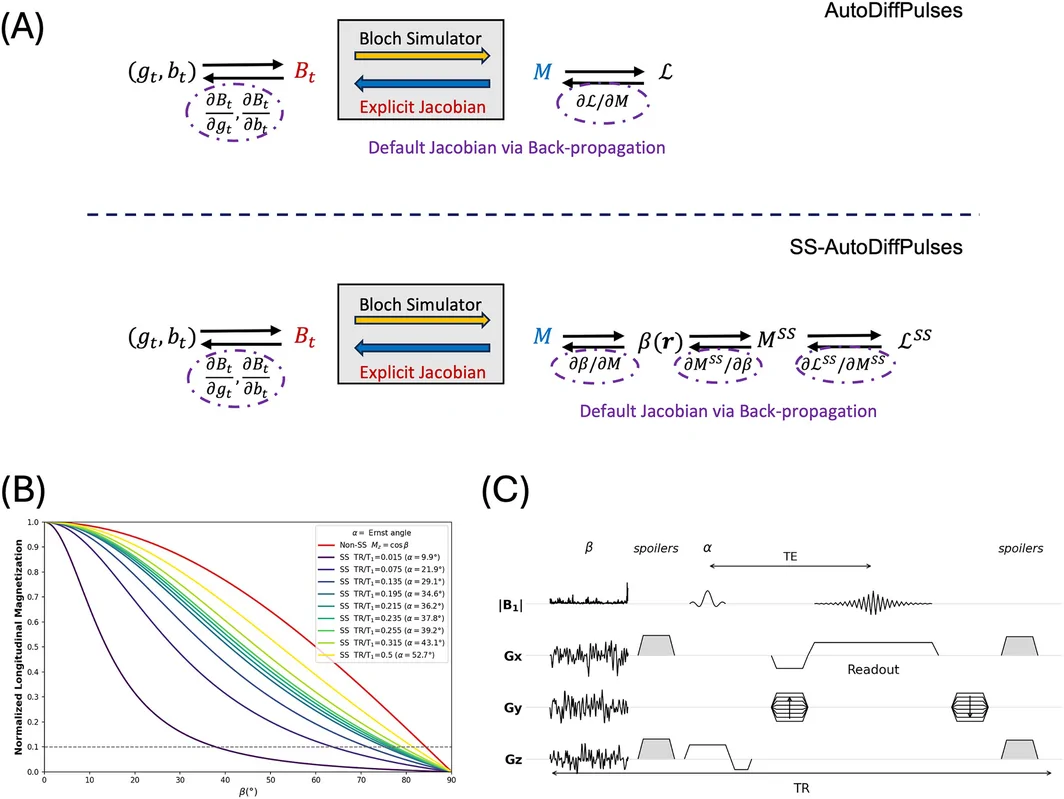
Comparison of the proposed SS‐ADP approach with the original ADP approach. (A) Flowcharts of the two frameworks. We incorporate the spin dynamics of the SS sequence into the forward process to correct the SS mismatch of the original framework. The SS magnetization $M^{\mathrm{SS}}$ is explicitly calculated from the output of the Bloch simulation M and is used to define the objective function. In the back‐propagation stage, the Jacobian of the Bloch simulator is explicitly defined as in Luo et al. [8] while the other derivatives are obtained from back‐propagation. (B) Normalized LM of the SS and non‐SS sequences versus saturation angle β. The non‐SS LM is calculated as $M_{z}=M_{0}\cos\left(\beta \right)$, and the SS LM is calculated based on Equation (3), with α equals to Ernst angle, at different $\mathrm{TR}/T_{1}$ ratios. Both curves are normalized at β=0. The 10% saturation is marked with a dashed line. (C) Timing diagram of the spoiled‐GRE SS sequence. An OVS/IVS pulse (β) is inserted before the excitation pulse (α).

#### 2.1.1 Review of AutoDiffPulses

In the original ADP framework, the gradient $g\in {\mathbb{R}}^{n_{T}\times 3}$ and RF $b\in {\mathbb{C}}^{n_{T}}$ waveforms are the variables to be optimized. From the gradient and RF waveforms, we can calculate the time‐dependent effective magnetic field $B_{t}$. With $B_{t}$, the Bloch simulator can simulate the evolution of the spins to the state at time T, that is, M; M is then compared with the target profile $M_{\mathrm{tgt}}$ to obtain a user‐defined loss L that can take arbitrary form. For example, one can define the loss to be the mismatch between the longitudinal components of M and of the target $M_{\mathrm{tgt}}$, plus a SAR (RF power) penalty:

(1) $$L={\left\|M_{z}-M_{\mathrm{tgt},z}\right\|}_{W}^{2}+\lambda {\left\|b\right\|}^{2}$$

where W is the spatial weighting function, and λ is the RF power penalty coefficient. The weighted‐*l*_2_‐norm ${\left\|\cdot \right\|}_{W}^{2}$ is defined as ${\left\|v\right\|}_{W}^{2}≔\sum_{i=1}v_{i}\cdot W_{i}$. The framework is implemented in Pytorch [9], which is an auto‐differentiation [10]‐based tool. With such a tool, simple Jacobians such as $\partial L/\partial M_{T}$ and ∂B/∂g,∂B/∂b, will be automatically computed for later use in the gradient‐descent‐like optimization. For the most computationally‐heavy process, that is, Bloch simulation, as in Luo et al. [8] we substitute the default Jacobian with an explicitly defined one to save computation overhead.

#### 2.1.2 Novelty of SS‐ADP

The main contribution of our SS‐ADP framework is to explicitly incorporate the SS calculation into the forward process. Specifically, we derived the formula that describes the relationship between the SS magnetization $M^{\mathrm{SS}}$ and the spatially‐selective suppression pulse. Consider an RF‐spoiled GRE sequence (e.g., SPGR/FLASH/T1‐FFE) as shown in Figure 1C, where a spatially‐selective pulse β is inserted before the signal excitation pulse α to suppress the unwanted signal. We can calculate the suppression flip‐angle β(r) from the output of the Bloch simulation M(r) as follows:

(2) $$\beta \left(r\right)=\arctan\frac{|M_{\mathrm{xy}}\left(r\right)|}{M_{z}\left(r\right)}$$

One can derive the formula for SS magnetization as follows:

(3) $$M^{\mathrm{SS}}\left(r\right)=\frac{M_{0}\left(1-E_{1}\right)\cdot \text{cosβ}\left(r\right)}{1-\cos\beta \left(r\right)\cos\alpha \cdot E_{1}}\cdot \left[0,0,1\right]≔|M^{\mathrm{SS}}\left(r\right)|\cdot \left[0,0,1\right]$$

assuming initial magnetization is along the longitudinal axis, where $M_{0}$ is the initial LM, $E_{1}≔e^{-\mathrm{TR}/T_{1}}$ is the $T_{1}$ relaxation term, and α is the excitation angle. The SS magnetization $M^{\mathrm{SS}}$ is defined at the moment right before the α pulse. Figure 1B shows the longitudinal component of $M^{\mathrm{SS}}$ versus β, for different $\mathrm{TR}/T_{1}$ ratios. The SS target pattern is defined as:

(4) $$M_{\mathrm{tgt}}^{\mathrm{SS}}\left(r\right)=\frac{M_{0}\left(1-E_{1}\right)\cdot \cos\beta_{\mathrm{tgt}}\left(r\right)}{1-\cos\beta_{\mathrm{tgt}}\left(r\right)\cos\alpha \cdot E_{1}}\cdot \left[0,0,1\right]$$

where $\beta_{\mathrm{tgt}}\left(r\right)$ is the user‐defined target suppression flip‐angle. Similar to the original ADP, one can choose the form of the loss function, which typically consists of an error term and a penalty term. For the experiments we will show in the following sessions, we chose the loss function to be the mismatch between the resultant SS LM and target LM profiles, plus an SAR penalty:

(5) $$L^{\mathrm{SS}}={\left\|M_{z}^{\mathrm{SS}}-M_{\mathrm{tgt},z}^{\mathrm{SS}}\right\|}_{W}^{2}+\lambda {\left\|b\right\|}^{2}$$

We use the same waveform initialization and optimizer as in Luo et al. [8].

### 2.2 Experimental Setup

We used the SPGR sequence in Figure 1C to acquire MRI images. The sequence consists of three major parts: a spatially‐selective suppression pulse β, an excitation pulse α, and a readout segment. All experiments were carried out on either a GE MR750 3T or GE UHP 3T scanner, with Nova 32Ch head receive coil arrays and single‐channel (built‐in body coil) transmission.

To demonstrate the improved excitation accuracy of SS‐ADP, we carried out two experiments: OVS and IVS. OVS pulses can be used in rFOV imaging [11] to allow fewer phase encoding lines to be acquired. IVS can be used to suppress unwanted physiological fluctuations, for example, CSF in the central ventricles in brain MRI. It has been reported [12] that IVS can reduce the parallel imaging g‐factor and increase SNR within the ROI. The excitation NRMSEs of the ADP and the SS‐ADP frameworks were evaluated.

We also investigated the effects of OVS pulses on improving accelerated imaging. Specifically, we examined OVS pulse's effect on reducing acceleration aliasing within the ROI in stack‐of‐spiral (SOSP) rFOV imaging.

The type of sequences used in this paper are summarized as follows:

1. Box‐shaped OVS: Here, we design a 2D OVS pulse that selectively suppresses the in‐plane signal, for a square ROI with side length FOV/3. We used $T_{1}=1470\;\mathrm{ms}$ in the designs. We used a slab‐selective excitation α pulse to restrict the FOV in the through‐plane dimension. The slab thickness is 0.35*FOV_z_. We implemented two different acquisition schemes:
  - 3D spin warp readout. Matrix size 60×60×60. Resolution is 4mm isotropic. FOV = [24, 24, 24] cm, TR = 80 ms, FA = 20° (Protocol 1).
  - 3D‐EPI readout. An $R_{y}\times R_{z\Delta z}=1\times 6_{z2}$ blipped‐CAIPI [13, 14] 3D‐EPI acquisition is used. Matrix size 90×90×60, resolution is 2.4mm isotropic, FOV = [21.6,21.6,14.4]cm, TR = 80 ms, FA = 20° (Protocol 2).
2. Cylinder‐shaped OVS: The ROI is a cylinder with diameter equals FOV/3. A multi‐shot SOSP acquisition with 3 arms is used. Sequence parameters: matrix size 100×100×60, resolution 2.4mm isotropic, FOV = [24,24,14.4]cm, TR = 50 ms, FA = 12°. In the RF design, we used $T_{1}=500\;\mathrm{ms}$ for phantom experiments and $T_{1}=1470\;\mathrm{ms}$ for in vivo experiments (Protocol 3).
3. Sphere‐shaped IVS: Here, the IV is a centered sphere with radius 2.4 cm. Excitation slab thickness is 0.8*FOV_z_. We used $T_{1}=1470\;\mathrm{ms}$ in the designs. We acquired the data with the same readout as in Protocol 2 (Protocol 4).

For all saturation pulses, the target flip angle in the saturation regions is $90^{\circ }$.

## 3 Results

### 3.1 ADP Versus SS‐ADP: Excitation Accuracy

Phantom OVS results are shown in Figure 2A. The SS‐ADP design produces an excitation pattern that better matches the target compared to the ADP design. It is noteworthy that SS‐ADP OVS has higher residual signal in the OV. This is because in non‐SS design, $M_{z}$ has a steep derivative R β around β=90° (Figure 1B), which makes ADP stricter on β variation in OV. Despite this, SS‐ADP achieves lower excitation NRMSE, with a more accurate IV shape and improved IV signal preservation. The ADP pulse suppresses some of the signal inside the ROI, making the achieved SS excitation smaller than desired.

**FIGURE 2:**
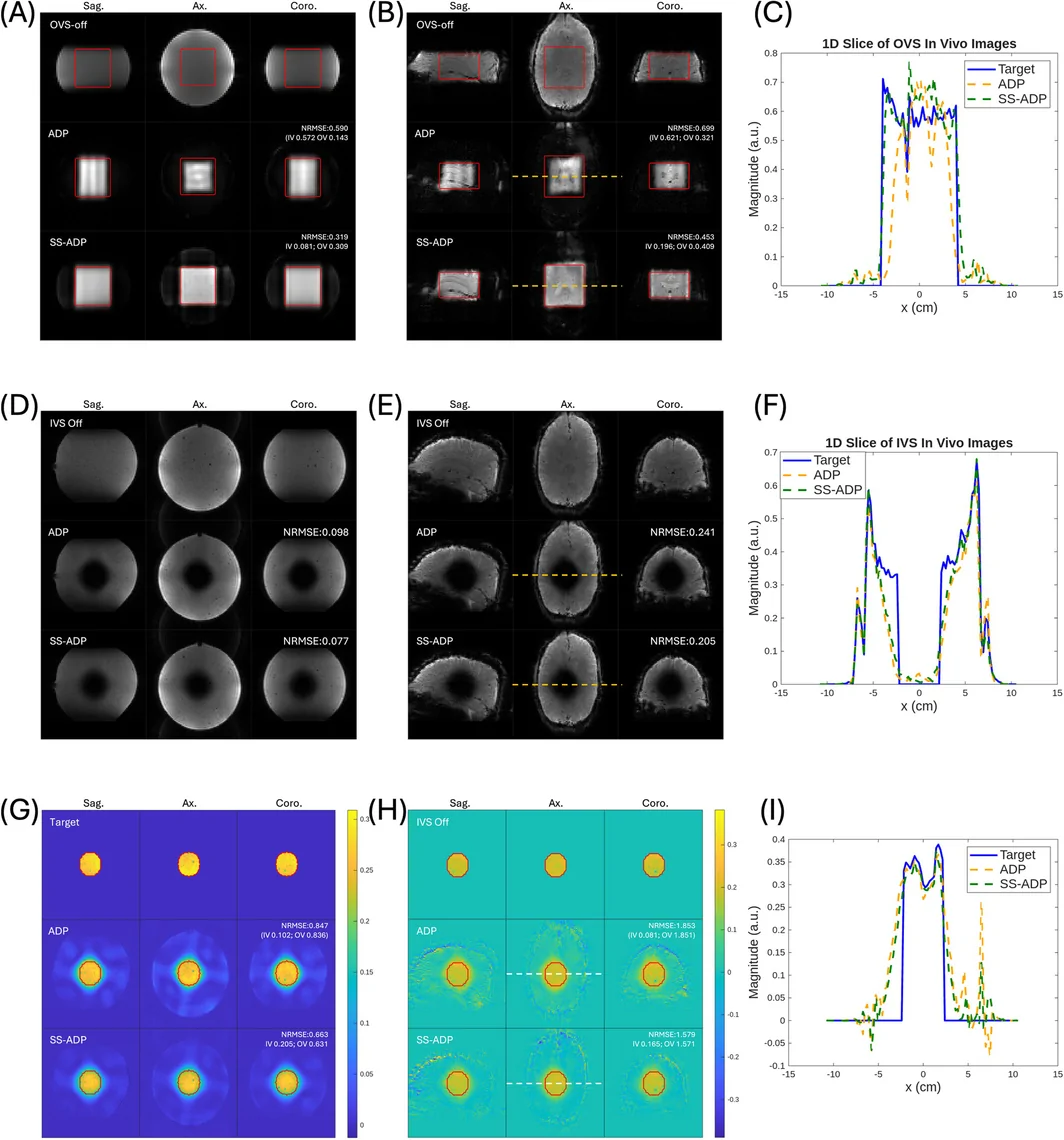
OVS and IVS experimental results using tailored RF pulses designed with ADP and SS‐ADP algorithms. (A) Phantom OVS imaging results (Protocol 1, c.f. Section 2.2). Shown are normalized images of: OVS‐off (top), OVS with ADP (middle), and OVS with SS‐ADP (bottom). The target is the OVS‐off images masked by the ROI. The ROI is marked by the red box. The overall excitation NRMSE, and the NRMSE within IV/OV are marked at the top‐right corners of each row. (B) Corresponding in vivo OVS imaging results (Protocol 2). (C) 1D profiles of the target and two OVS images corresponding to the yellow dashed lines in (B). The SS‐ADP design has a FWHM closer to the target and the IV signal is better preserved. ADP OVS results in over‐saturation because the non‐SS model does not account for the short RF repetition time. (D and E) IVS phantom (D) and in vivo (E) experiment results (Protocol 4): Normalized images without IVS (top), and with IVS pulses designed using ADP (middle) or SS‐ADP (bottom). (F) 1D profiles corresponding to the target (the IV signal of the IVS‐off images is set to zero as target), and the yellow dashed lines in (E). The 1D profiles look similar for both designs in the IVS case due to the use of “don't‐care” region. (G and H) Magnitude subtraction between IVS‐off and IVS‐on images for ADP (middle) and SS‐ADP (bottom) for the images in (D and E). Target is cropped from the IVS‐off images. (I) 1D profiles along the white dashed lines in (H).

Figure 2B presents the results of the in vivo OVS experiment. Here, the SS‐ADP design better preserves both ROI shape and signal uniformity. The corresponding 1D profiles, taken along the yellow‐dashed lines in Figure 2B, are shown in Figure 2C and further highlight the closer agreement between the SS‐ADP profile and the target.

Figure 2D–F summarize the IVS phantom and in vivo experiment results. In both cases, SS‐ADP reduces the NRMSE relative to the ADP pulse. Note that in the IVS example, SS‐ADP produces a similar spread of the saturation region as ADP. This is because we defined a “don't‐care” region at the IV/OV boundary to prevent the transition band from dominating the optimization. In the OVS case, we did not use such a “don't‐care” region because preserving target IV is more critical there: it is important to not destroy signal within target IV in rFOV imaging. The use of “don't‐care” region may lead to an apparent spread of the saturation region in the SS‐ADP case. This makes the SS‐ADP lose some of the advantage of preventing over‐saturation. However, its advantage of reduced perturbation in the non‐saturation region (i.e., OV) is still present. To make this more visually clear, the magnitude subtraction between IVS‐off and IVS‐on images are shown in Figure 2G–I. SS‐ADP has a higher IV excitation error but a lower OV error, demonstrating again that it better preserves the ROI signal.

### 3.2 Error as a Function of $\mathrm{TR}/T_{1}$ Ratio

To determine what $\mathrm{TR}/T_{1}$ range over which SS‐ADP can provide clear advantages over ADP, we designed OVS pulses for $\mathrm{TR}/T_{1}$ ratios ranging from 0.015 to 0.335, with (1) fixed $T_{1}=1500\mathrm{ms}$, and (2) fixed TR=50ms. The loss functions does not include SAR penalty in either design. This prevents the complication of setting up different λ values to account for the scale difference in the excitation error terms of Equations (1) and (5). For each (TR, $T_{1}$) pair, we performed Bloch simulations accounting for $\mathrm{TR}-T_{1}$‐dependent SS effects. The excitation angle α was set to the corresponding Ernst angle to maximize IV signal. We evaluated excitation accuracy using 1D and 2D profiles, quantified by the NRMSEs, and the full‐width‐half‐maximum (FWHM) of the 1D slice. Figure 3 shows the results of these SS simulations with varying $\mathrm{TR}/T_{1}$ ratios. At very small $\mathrm{TR}/T_{1}$ ratio, the perturbation of the IV signal in ADP becomes exaggerated because the pulse is repeated very rapidly. This results in a smaller than expected FWHM. As $\mathrm{TR}/T_{1}$ increases, the ADP SS excitation pattern in the IV gets closer to the target. While the gross excitation NRMSE of ADP gets smaller than the SS‐ADP for $\mathrm{TR}/T_{1}>0.175$ (Figure 3A), ADP has a consistently higher IV NRMSE (Figure 3B) and lower OV NRMSE (Figure 3C) than those of SS‐ADP for all the simulated ratios. This is because in ADP, $M_{z}$ has a larger absolute derivative near β=90°, making it more sensitive to OV β variations (Figure 1B). In contrast, $M_{z}^{\mathrm{SS}}$ is steepest near β=0°, so it is more sensitive to IV β fluctuations. The difference in IV NRMSE becomes relatively small above $\mathrm{TR}/T_{1}\approx 0.2$. Based on these simulations, we suggest that SS‐ADP should be used for SS sequences with $\mathrm{TR}/T_{1}≲0.2$, otherwise one may choose SS‐ADP or ADP depending on whether IV or OV excitation accuracy is more desirable for a given application. Note that in these SAR‐penalty‐free designs, the SS‐ADP pulses have smaller pulse‐average‐power (PAP) than the ADP pulses up until $\mathrm{TR}/T_{1}≳0.135$ (Figure 3E).

**FIGURE 3:**
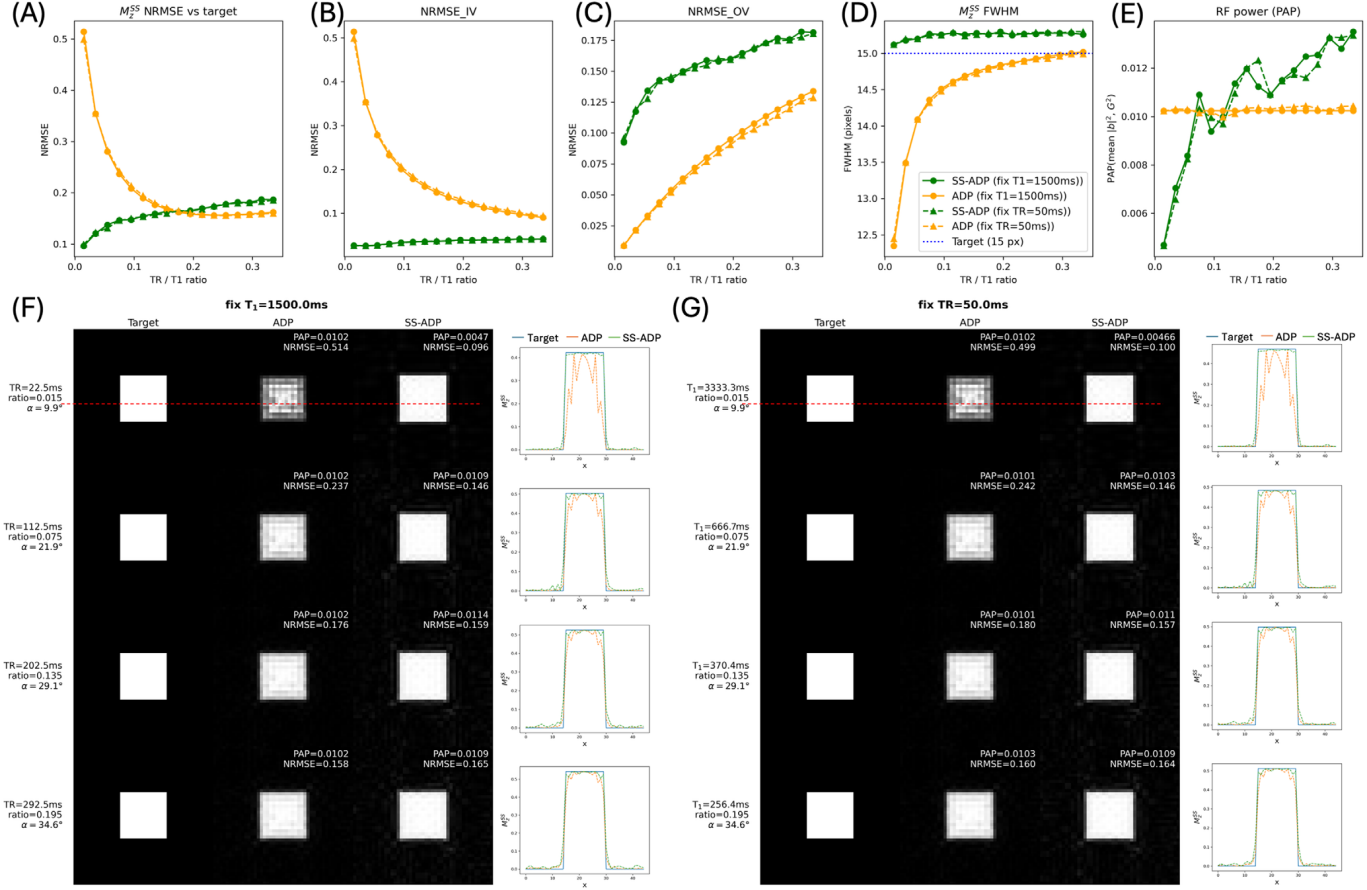
Simulations of SS‐ADP and ADP 2D OVS designs with varying $\mathrm{TR}/T_{1}$ ratios. The spatial simulation domain is two‐dimensional with matrix size 45×45, FOV = 24 × 24 cm. (A–C) NRMSE evaluated over (A) full FOV, (B) IV, and (C) OV, for ADP (orange) and SS‐ADP (green), over two types of $\mathrm{TR}/T_{1}$ sweeps: Fixed $T_{1}$ (circles) and fixed TR (triangles). (D) The FWHM of the 1D profiles in (F and G). The target FWHM is 15 pixels. The 1D profile is interpolated to a grid size of 0.01 pixels before FWHM calculation. (E) The RF power of both designs across $\mathrm{TR}/T_{1}$ ratios. (F and G) SS LM patterns of target (left), ADP (middle), and SS‐ADP (right) pulses at some examplary $\mathrm{TR}/T_{1}$ ratios for (F) fixed $T_{1}=1500\;\mathrm{ms}$ and (G) fixed TR=50ms. The target pattern is calculated according to Equation (3). The NRMSE and PAP are marked at the top‐right corners. The NRMSE of the ADP patterns consistently decreases as TR increases whereas SS‐ADP NRMSE stays relatively constant. The 1D profiles along the red dashed line in (F and G) are shown on the right. The spatial weighting W is inversely proportional to the areas of IV and OV, that is W(IV):W(OV)=8:1 for all designs. SS‐ADP provides significantly improved SS excitation accuracy up to $\mathrm{TR}/T_{1}∼0.2$, and reduced SAR up to $\mathrm{TR}/T_{1}∼0.13.$ FWHM, Full‐width‐half‐maximum; LM, Longitudinal magnetization; PAP, Pulse‐average‐power.

To investigate the excitation profile in a non‐SS setting, we show the regular Bloch simulation (i.e., “single‐shot” simulation) result in Figure S1. ADP has smaller “single‐shot” excitation NRMSE than SS‐ADP. The IV fluctuation feature of the ADP in “single‐shot” simulation is similar to the one observed in SS simulation but less pronounced.

### 3.3 Trade‐Off Between Excitation Accuracy and SAR

In Equation (1), λ controls the trade‐off between excitation accuracy and RF energy. To investigate the impact of λ on this trade‐off, we designed cylindrical‐shaped OVS pulses with varying λ values and plotted the Pareto fronts of ADP and SS‐ADP designs. The results are shown in Figure 4. For $\mathrm{TR}/T_{1}≲0.2$, SS‐ADP has a better trade‐off than the ADP design (Figure 4A). This is because in the small‐TR regime, the SS $M_{z}$ can be well saturated with a flip angle much lower than $90^{\circ }$. For example, when $\mathrm{TR}/T_{1}=0.015$, SS $M_{z}$ can be suppressed below 10% with a saturation angle as low as $40^{\circ }$ (see Figure 1B), whereas the same percent of saturation in the non‐SS case entails a flip angle much closer to $90^{\circ }$. SS‐ADP loses the SAR reduction effect for $\mathrm{TR}/T_{1}$ ratios above 0.2 (Figure 4B), as the SS $M_{z}$ curves get closer to the non‐SS curve in this $\mathrm{TR}/T_{1}$ regime (Figure 1B). Note that Figure 4 is plotted by using the same initialization and fixed number of iterations. Thus, each point on the Pareto front is not necessarily the optimal solution due to possible non‐convexity and convergence issues.

**FIGURE 4:**
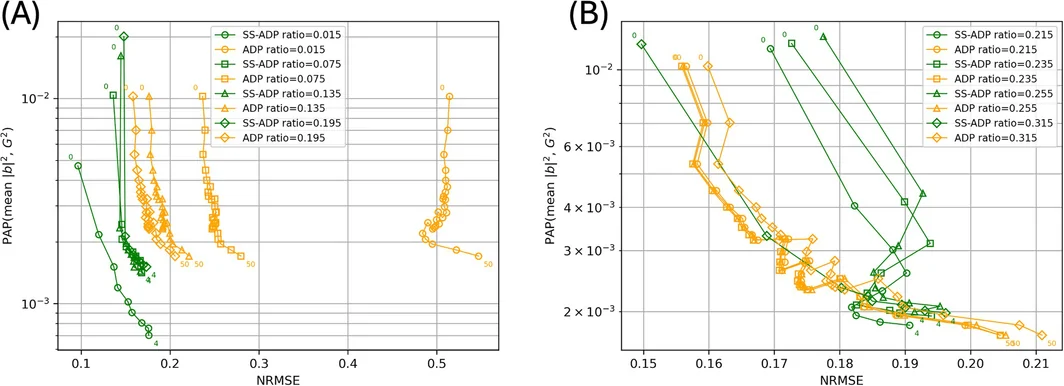
Pareto front of excitation accuracy and RF power for ADP and SS‐ADP, across different $\mathrm{TR}/T_{1}$ values. The numbers next to the dots at both ends of the curves indicate the range of the λ sweep. The horizontal axis is the normalized error between achieved SS LM profile $M_{z}^{\mathrm{SS}}$ and the target profile $M_{\mathrm{tgt},z}^{\mathrm{SS}}$, whereas the vertical axis shows the pulse‐average‐power (PAP). (A) For $\mathrm{TR}/T_{1}≲0.2$, the Pareto front of SS‐ADP is closer to the origin, meaning that to achieve the same NRMSE, SS‐ADP pulses have a smaller SAR. (B) However, for $\mathrm{TR}/T_{1}≳0.2$, ADP pulses provides a better trade‐off.

### 3.4 Improving rFOV Imaging

Figure 5A shows orthogonal views of the full‐FOV SOSP in vivo experiments with OVS‐off, ADP OVS, and SS‐ADP OVS. Here we use SOSP acquisition (Protocol 3) because both in‐plane axes will benefit from the 2D restricted FOV. There is noticeable residual OV signal at the air‐tissue boundary due to high off‐resonance in both OVS cases. A *B*_0_‐informed design reduces the OV energy from 549 to 482 (12%, a.u.) for ADP, and from 1187 to 1009 (15%, a.u.) for SS‐ADP. Figure 5B shows the rFOV imaging results. The OVS‐off image from (A) is cropped to the central 1/3 in‐plane FOV as a reference. The OVS images are acquired with in‐plane acceleration factor R=3. Comparing against ADP OVS, despite having higher OV energy, SS‐ADP reduces accelerated rFOV image error from 0.157 to 0.050 for *B*_0_‐informed design. This is because SS‐ADP better preserves the IV signal, as has been shown in the earlier sections.

**FIGURE 5:**
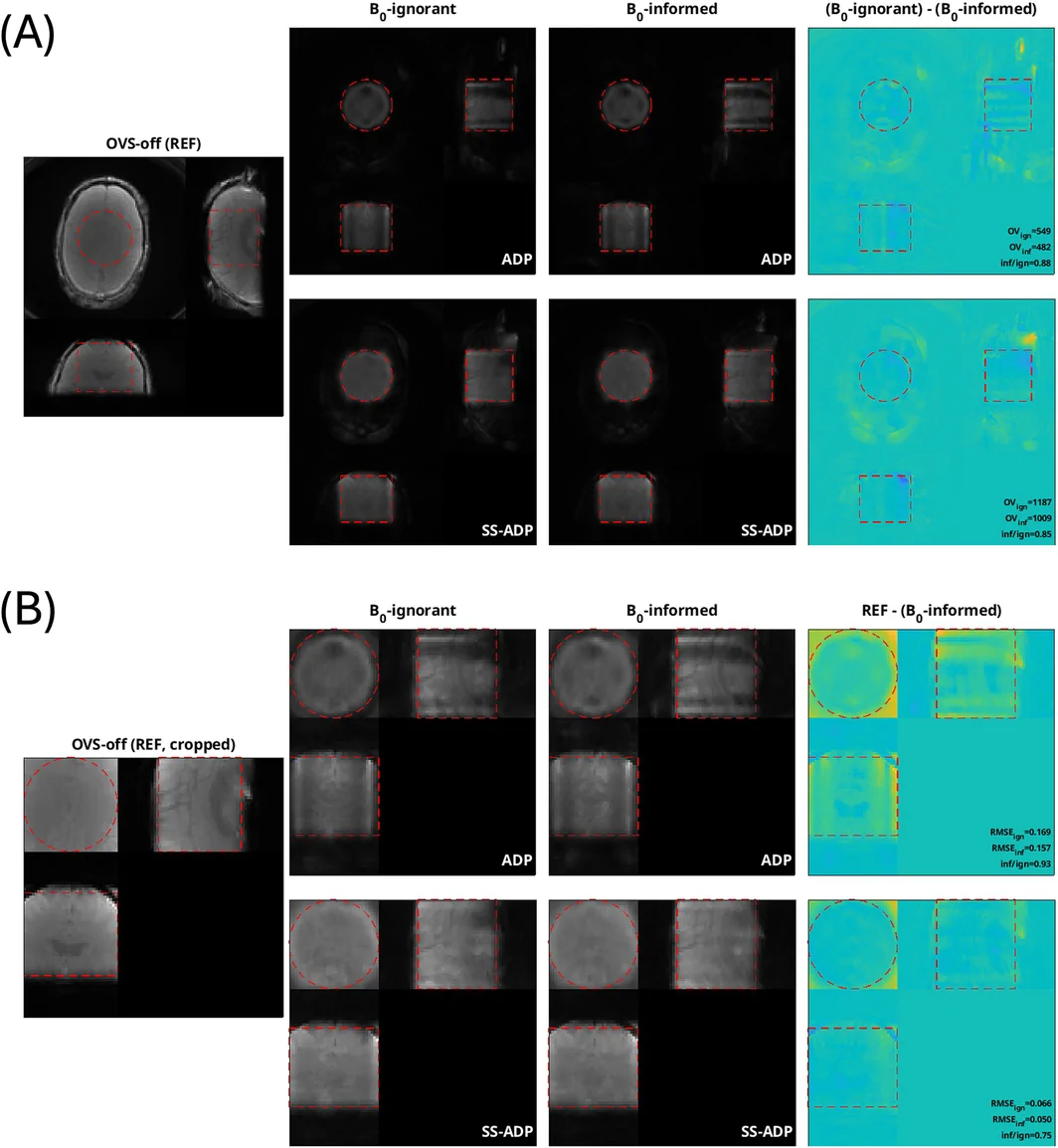
In vivo rFOV imaging using cylindrical‐shaped OVS pulses and SOSPs acquisition with 3 in‐plane spiral arms. (A) For reference, shown here are full‐FOV images without OVS (left), with ADP OVS (top row), and with SS‐ADP OVS (bottom row). Magnitude subtraction between *B*_0_‐ignorant and *B*_0_‐informed images (right) shows the reduction of OV energy. The OV energy of *B*_0_‐ignorant/informed and their ratio are marked at the lower‐right corner of the figures. (B) rFOV images without OVS (left), with ADP OVS (top row), and with SS‐ADP OVS (bottom row). The OVS‐off image in (A) is cropped to the central 1/3 in‐plane FOV to serve as the reference. The OVS images are acquired with prospective in‐plane acceleration factor R=3. The error between accelerated images with *B*_0_‐informed OVS and the reference is shown on the right. The IV RMSE for *B*_0_‐ignorant and *B*_0_‐informed accelerated images, and their ratio, are shown at the lower‐right corner of the figures. SS‐ADP reduces the acceleration error in both settings.

## 4 Discussion

We compared SS excitation accuracy for SS‐ADP and ADP in SPGR sequences with TR=80ms ($0.165T_{1}$ for the phantom and $0.055T_{1}$ for gray matter at 3T (Figure 2)). The proposed SS‐ADP framework reduces OVS excitation NRMSE by 46% and 35% for phantom and in vivo experiments, respectively, compared to ADP. We acknowledge that the over‐saturated IV shape in ADP can be regained by using an artificially larger design target IV. However, this workaround can not remedy the fact that ADP pulses still leads to higher perturbation to the IV signal compared to SS‐ADP.

We note that the excitation accuracy depends on $T_{1}$ (Figure 3) value, thus, it is important to use $T_{1}$ values in the RF design that match the scan subject. The original ADP tool provides the option to include a $T_{1}$ map into the RF pulse optimization, and our SS‐ADP version inherits this capability. We did not use this feature in this work due to the lack of $T_{1}$ map information.

SS‐ADP pulses have lower RF power compared to ADP given similar excitation error, up to $\mathrm{TR}/T_{1}≃0.2$. This is particularly beneficial in short‐TR settings since SAR is a common constraint in short‐TR SS imaging.

The current SS‐ADP tool does not optimize the pulse duration. For example, the 2D OVS pulses used in this work had a pre‐determined duration of 4–6 ms (see Figure S2). The fact that the PAP of SS‐ADP pulses is relatively lower in the small $\mathrm{TR}/T_{1}$ regime indicates that there is room to reduce pulse duration. Incorporating time‐optimal design into SS‐APD is a future research direction.

Compared to ADP designs, the proposed SS‐ADP approach has a slightly higher computation overhead due to the additional SS‐equation computation. This is illustrated in Figure S3 which shows the convergence plot for one design example. For the same number of iterations, SS‐ADP takes longer to converge.

The framework could be extended to accommodate other types of SS sequences. For example, one could substitute Equation (3) with a modified version of Equation (2) in Scheffler et al. [15] to make SS‐ADP applicable to balanced‐SSFP sequences. The utility of the proposed SS‐ADP approach for non‐spoiled SS sequences is an open research question.

## 5 Conclusions

We adapted the auto‐differentiation‐based RF design tool from Luo et al. [8] to include the SPGR sequence signal equation (namely, SS‐ADP), and demonstrated its usage in designing OVS and IVS pulses in simulation, phantom, and in vivo experiments. We also demonstrated in a SOSP in vivo experiment that a (*B*_0_‐informed) SS‐ADP OVS pulse can reduce rFOV imaging acceleration error compared to ADP OVS pulses. The proposed SS‐ADP framework designs tailored RF pulses with improved overall excitation accuracy and lower SAR in SPGR sequences for $\mathrm{TR}/T_{1}≲0.2$ compared to the original ADP [8].

## Funding

This work was supported by the National Institutes of Health (NIH); Grant/Award Number: U24NS120056.

## Conflicts of Interest

The authors declare no conflicts of interest.

## Supporting information


**Figure S1:** Regular Bloch (i.e., “Single‐shot”) simulation (A) Target “single‐shot” LM $M_{\mathrm{tgt},z}$ (top) and “single‐shot” LM for ADP pulse (bottom). Here, simulation matrix size is 45×45, FOV = [24 cm, 24 cm]. IV is a square with side length 1/3 of the FOV. $M_{\mathrm{tgt},z}=1$ for IV, $M_{\mathrm{tgt},z}$ = 0 for OV. The spatial weighting in loss function is W(IV):W(OV)=8:1. (B) “Single‐shot” simulation of SS‐ADP OVS pulses designed with $T_{1}=1500\;\mathrm{ms}$, and TR = $0.015T_{1},0.075T_{1},0.135T_{1},0.215T_{1}$. The excitation NRMSE are marked at the lower‐right corner of each panel. As TR increases in the design, the excitation replySS‐ADP gets closer to that of ADP. (C) 1D excitation profiles intersected at the mid‐line of the 2D profiles in (A and B). SS‐ADP pulses in general have more uniform pass‐band but have worse stop‐band than ADP, especially when design $\mathrm{TR}/T_{1}$ is small.
**Figure S2:** RF pulse waveforms of 2D OVS pulses Top to bottom: RF amplitude, RF phase, and gradient waveforms of the initalization (blue), ADP design (orange), and SS‐ADP design (green) for the 2D OVS example. The initialization is from the “extended‐kt point” method with a duration of 6.28 ms. The gradient waveforms of both ADP and SS‐ADP designs stay very close to the initialization.
**Figure S3:** Convergence rate of ADP and SS‐ADP optimizations for the 2D OVS design. The loss function is normalized to the initial loss. Here, number of iteration is 8 for both designs. SS‐ADP takes slightly longer time to converge.

## Data Availability

The codebase of the SS‐ADP tool can be found at https://github.com/tianrluo/AutoDiffPulses.git.
