# Supplementary material for: Improved Accuracy of Multidimensional Spatially‐Selective Saturation RF Pulses for Short‐Repetition‐Time Steady‐State Imaging
Source: Magn Reson Med. 2026 Aug 26;97(1):250–8. doi: 10.1002/mrm.70573 (PMC13746087; doi:10.1002/mrm.70573)
Supplement: Supplementary file 1 [file MRM-97-250-s001.zip › MRM_YHe_2026_revision1_SI_clean/supporting_information_rev.pdf]

Improved Accuracy of Multidimensional Spatially-Selective Saturation RF Pulses for Short-Repetition-Time Steady-State Imaging

Yongli He, Douglas C. Noll, Jon-Fredrik Nielsen
University of Michigan

Supporting Information

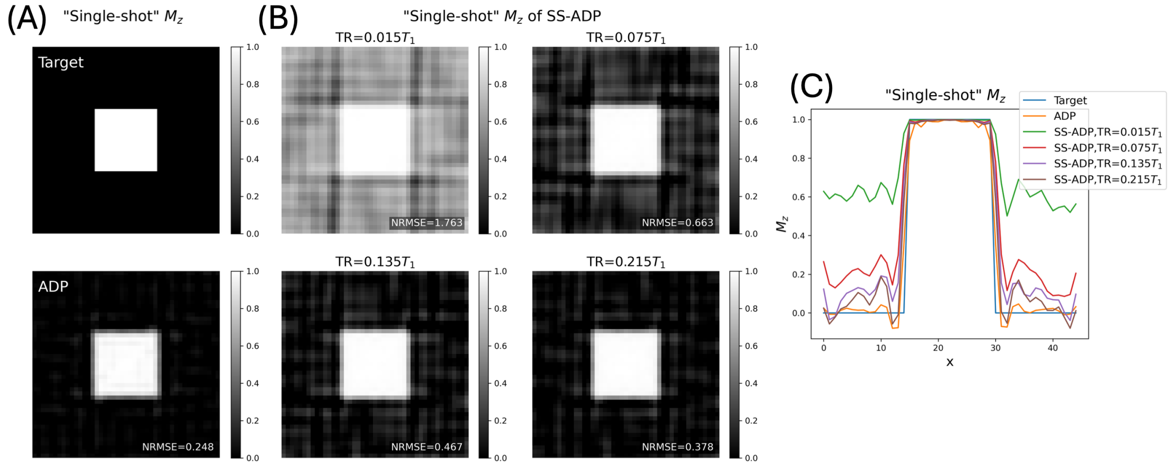

FIGURE 1 Regular Bloch (i.e., "Single-shot") simulation (A) Target "single-shot" LM $M_{tgt,z}$ (top) and "single-shot" LM for ADP pulse (bottom). Here, simulation matrix size is 45×45, FOV=[24cm,24cm]. IV is a square with side length 1/3 of the FOV. $M_{tgt,z} = 1$ for IV, $M_{tgt,z}$=0 for OV. The spatial weighting in loss function is $W(\text{IV}) : W(\text{OV}) = 8 : 1$. (B) "Single-shot" simulation of SS-ADP OVS pulses designed with $\text{T}_1 = 1500$ ms, and $\text{TR} = 0.015\text{T}_1, 0.075\text{T}_1, 0.135\text{T}_1, 0.215\text{T}_1$. The excitation NRMSE are marked at the lower-right corner of each panel. As TR increases in the design, the excitation replySS-ADP gets closer to that of ADP. (C) 1D excitation profiles intersected at the mid-line of the 2D profiles in (A-B). SS-ADP pulses in general have more uniform pass-band but have worse stop-band than ADP, especially when design TR/T_1 is small. R2.C4

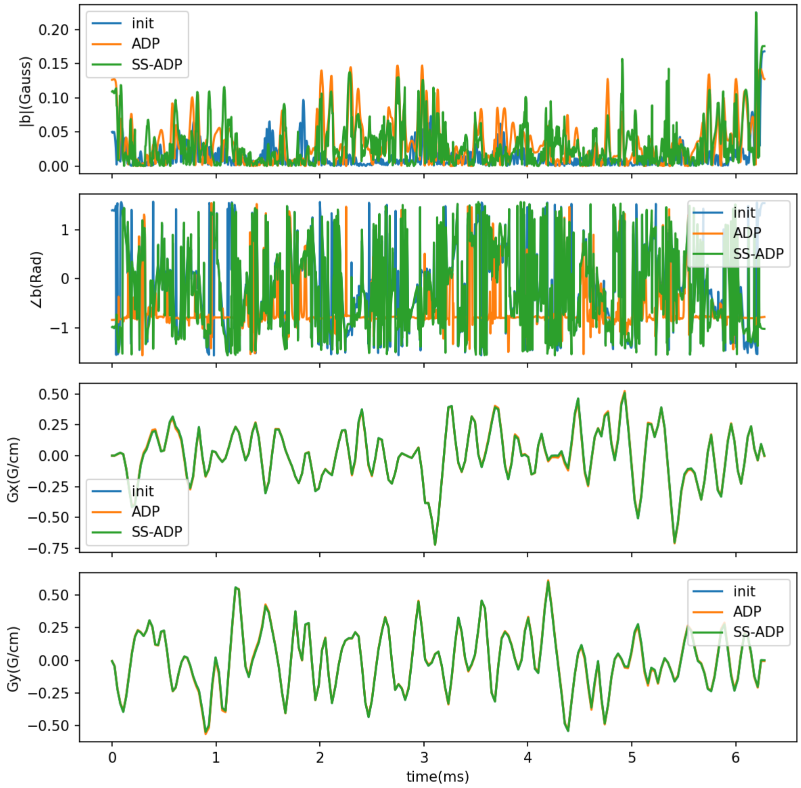

FIGURE 2 RF pulse waveforms of 2D OVS pulses Top to bottom: RF amplitude, RF phase, and gradient waveforms of the initalization (blue), ADP design (orange), and SS-ADP design (green) for the 2D OVS example. The initialization is from the "extended-kt point" [?] method with a duration of 6.28ms. The gradient waveforms of both ADP and SS-ADP designs stay very close to the initialization.

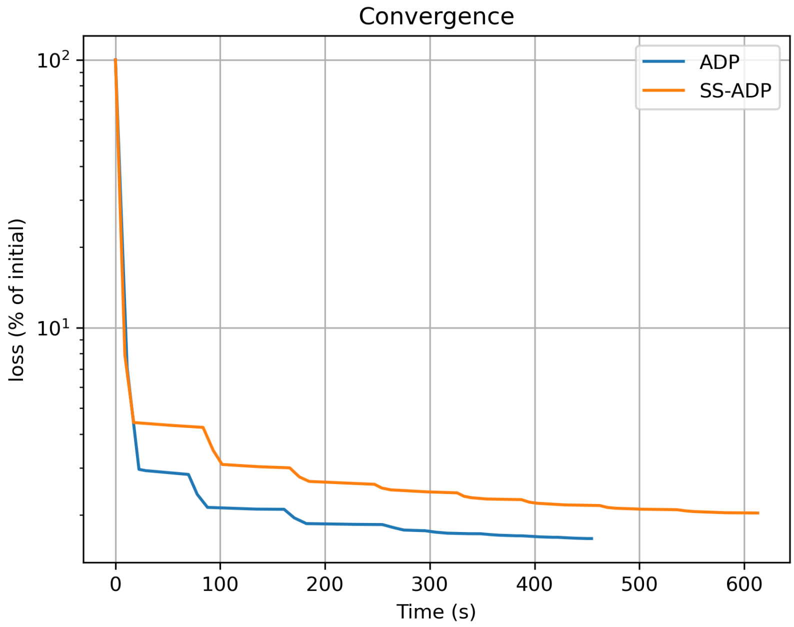

FIGURE 3 Convergence rate of ADP and SS-ADP optimizations for the 2D OVS design. The loss function is normalized to the initial loss. Here, number of iteration is 8 for both designs. SS-ADP takes slightly longer time to converge.
